# Supplementary material for: The Selection of Antibiotic- and Bacteriophage-Resistant Pseudomonas aeruginosa Is Prevented by Their Combination
Source: Microbiol Spectr. 2022 Sep 22;10(5):e02874-22. doi: 10.1128/spectrum.02874-22 (PMC9602269; doi:10.1128/spectrum.02874-22)
Supplement: Supplemental file 1 — Supplemental material. Download spectrum.02874-22-s0001.pdf, PDF file, 0.6 MB [file spectrum.02874-22-s0001.pdf]

Supplementary information to Ferran *et al.*

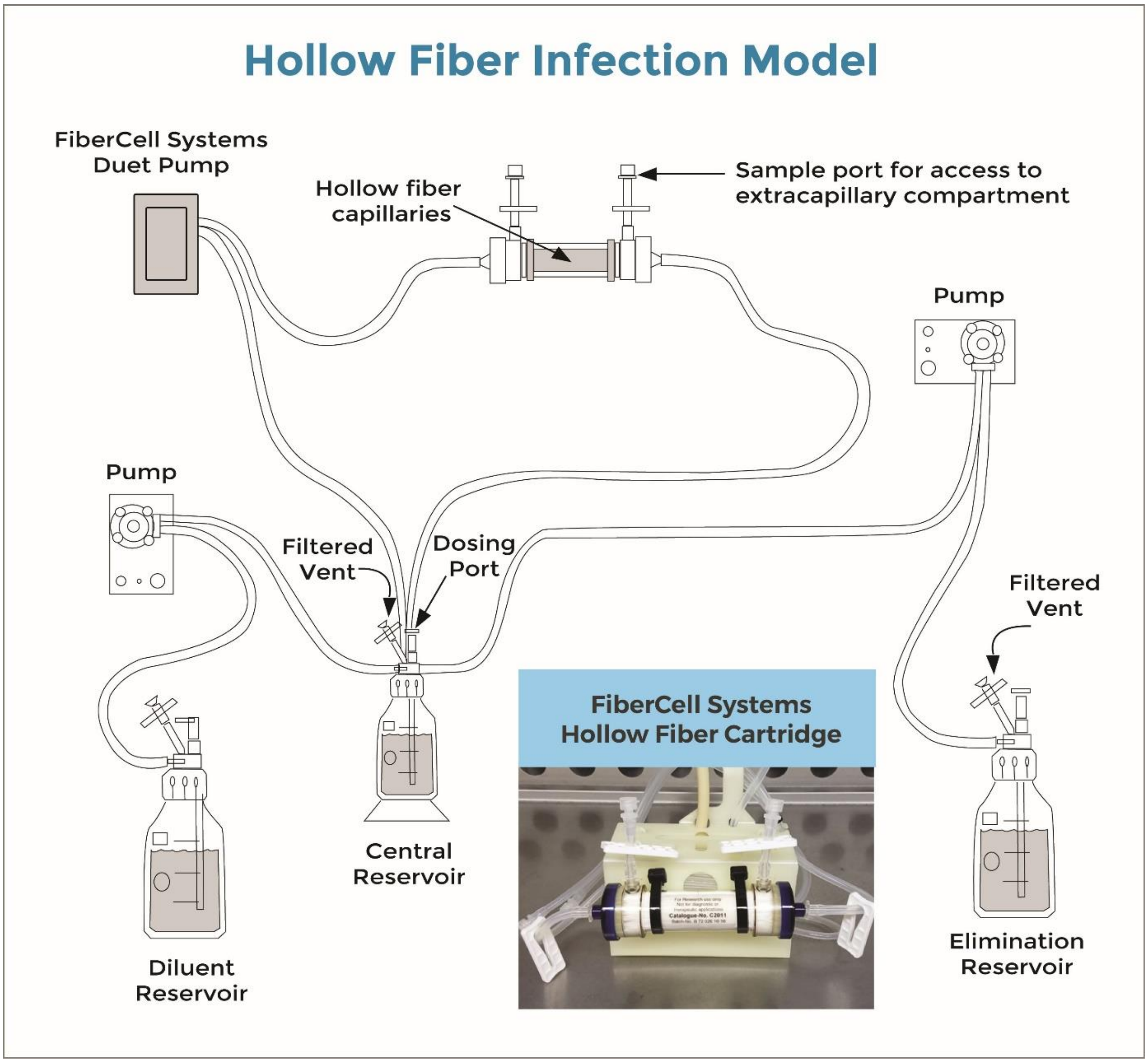

Figure S1. Schematic representation of the Hollow Fiber Infection Model kindly provided by FiberCell Systems®. Bacteria and phages were trapped in the extracapillary space of the cartridge (peripheral compartment) (see also embedded photo). Ciprofloxacin was added to the central reservoir and freely circulated through the cartridge and bacteria by means of the Fibercell Systems Duet pump® (FiberCell Systems, Inc., Frederick, MD, USA). Ciprofloxacin concentrations decreased over time after drug administrations, due to the continuous addition of a diluent (MHB) by means of another set of pumps

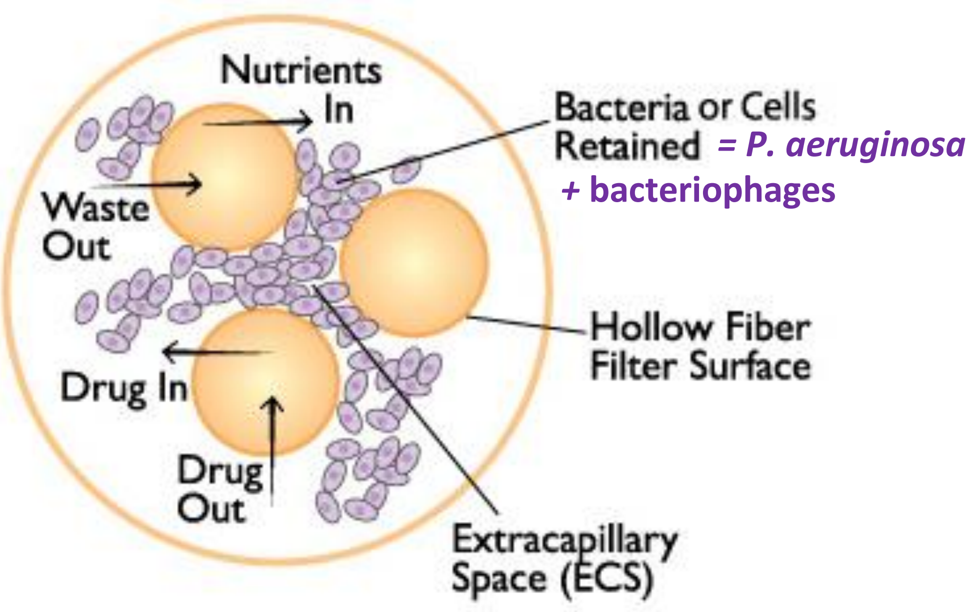

Figure S2. Schematic representation of the cross-section of the cartridge of the HFIM, kindly provided by FiberCell Systems®. Phages were directly introduced in the extracapillary space containing *P. aeruginosa* to simulate local administration. Both phages and *P. aeruginosa* remained trapped in this extracapillary space during the experiments. The drug, here ciprofloxacin, freely circulates through the fibers and was distributed both in the central reservoir and the extracapillary spaces of the cartridge.

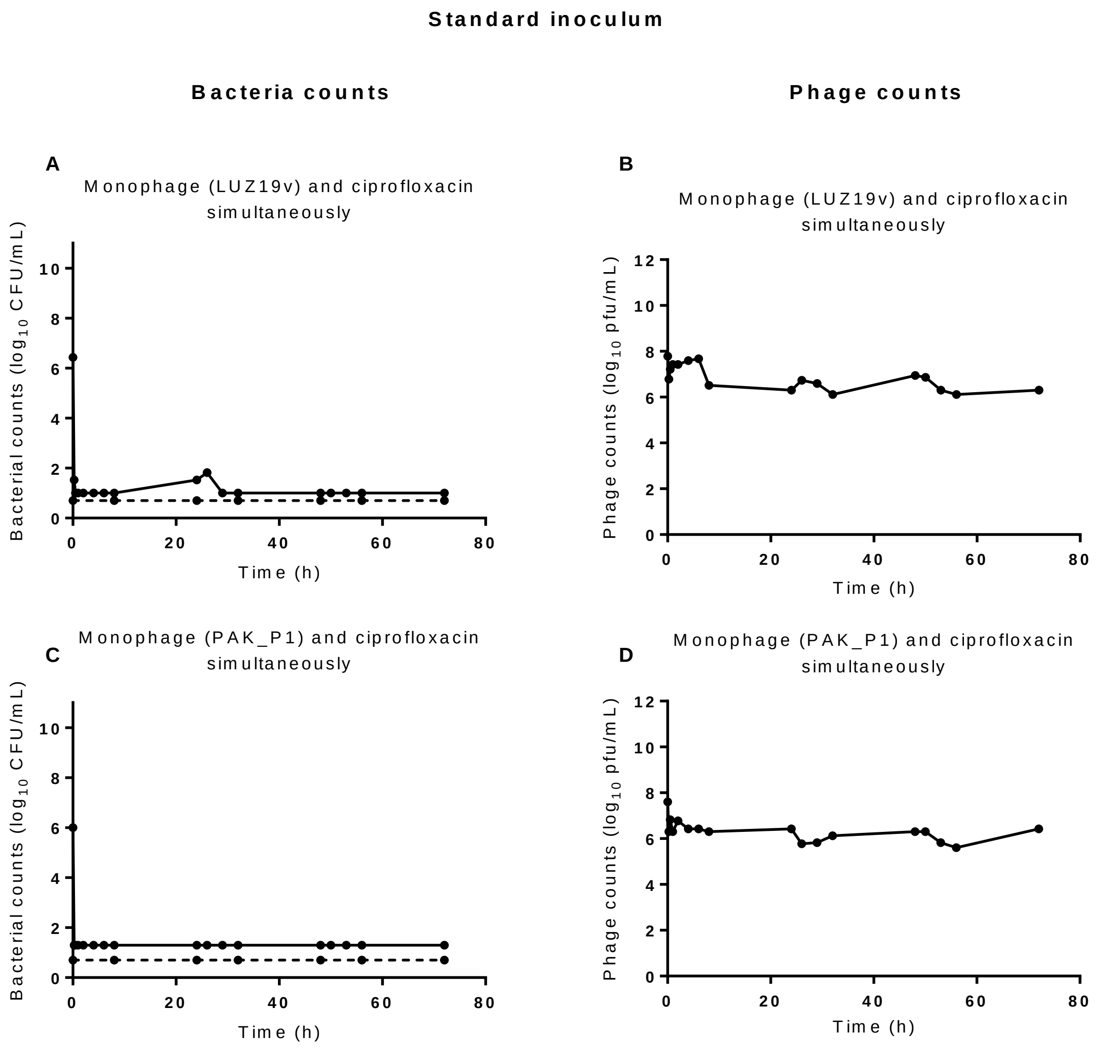

Figure S3. The concentration of *P. aeruginosa* strain PAK (Bacteria counts in $\log_{10}$ CFU/mL) and of phages (Phage counts in $\log_{10}$ pfu/mL) in the HFIM from 1 h post-inoculation to 72 h after exposure to the combination of ciprofloxacin and a single phage. A and B, combination of simultaneous administration of ciprofloxacin with LUZ19v (n=1). C and D, combination of simultaneous administration of ciprofloxacin with PAK_P1 (n=1). Solid lines represent total bacterial populations or total phage populations and dashed lines represent less-susceptible bacteria growing on agar containing 0.5 µg/mL of ciprofloxacin. Square and circles represent independent experiments. The limit of detection (LOD) was 1.5 $\log_{10}$ CFU/mL for bacteria and 1.5 $\log_{10}$ pfu/mL for phages.
